# Safety and efficacy of tezepelumab vs. placebo in adult patients with severe uncontrolled asthma: a systematic review and meta-analysis

**DOI:** 10.1038/s41598-022-24763-9

**Published:** 2022-12-03

**Authors:** Mahmoud Shaban Abdelgalil, Asmaa Ahmed Elrashedy, Ahmed K. Awad, Eman Reda Gad, Mahmoud M. Ali, Ramadan Abdelmoez Farahat, Bassant Hassan Shawki, Mohamed Abd-ElGawad

**Affiliations:** 1 Faculty of Medicine, Ain-Shams University, Cairo, Egypt; 2 Faculty of Medicine, Kafrelsheikh University, Kafrelsheikh, Egypt; 3 Faculty of Medicine, Cairo University, Cairo, Egypt; 4 Faculty of Pharmacy, Al-Azhar University, Assiut, Egypt; 5 Faculty of Medicine, Fayoum University, 5 Al-Touba Street, From Al-Fanya Street, Al-Hadka Road, Fayoum, Egypt

**Keywords:** Diseases, Health care, Medical research

## Abstract

Patients with severe uncontrolled asthma still experience acute asthma symptoms and exacerbations, particularly those with non-eosinophilic inflammation who take the maximum amount of standard drug therapy. Tezepelumab, a human monoclonal antibody, can improve lung function and enhance control of asthma symptoms in those patients, regardless of the disease’s baseline characteristics. This study aims to investigate the safety and efficacy of using tezepelumab in controlling severe symptoms of uncontrolled asthma. We performed a comprehensive literature search in several databases, including PubMed, Scopus, Web of Science, Cochrane Library, and clinicaltrial.gov, using a well-established search strategy to include all relevant publications. According to our inclusion criteria, we searched for randomized controlled trials comparing tezepelumab versus placebo in patients with severe, uncontrolled asthma. We analyzed the data using The Revman 5.4 program software. The search identified 589 potential articles. After excluding studies inconsistent with selection criteria, four studies were included and analyzed qualitatively and quantitatively. The pooled effect demonstrated the better performance of tezepelumab over the placebo regarding the decrease in annualized asthma exacerbation rate (MD = − 0.74, (95% CI [− 1.04, − 0.44], p < 0.00001)), asthma control questionnaire-6 (ACQ-6) Score MD = − 0.32, (95% CI [− 0.43, − 0.21], p < 0.00001)), blood eosinophil count (MD = − 139.38 cells/mcL, (95% CI [− 150.37, − 128.39], p < 0.00001)), feNO (MD = − 10 ppb, (95% CI [− 15.81, − 4.18], p = 0.0008)) and serum total IgE (MD = − 123.51 UI/ml, (95% CI [− 206.52, − 40.50], p = 0.004)). All tezepelumab groups had higher pre-bronchodilator forced expiratory volume in 1 s than the placebo group (MD = 0.16, (95% CI [0.10, 0.21], p < 0.00001)). Higher efficacy and safety profile was detected for tezepelumab to control the exacerbations of severe uncontrolled adult asthmatics.

## Introduction

According to the Global Initiative of Asthma (GINA), asthma is a condition that affects the lower parts of the airway, represented by recurrent respiratory manifestations including wheezing, breathlessness, tightness of the chest, and coughing as well as fluctuating airflow restriction^1^. In adults, the prevalence rate of asthma is estimated to be 4.5%, which translates to nearly 300 million persons with asthma globally. In developed countries, this prevalence reaches 21.5%^2^.

In most cases, airway restriction and asthmatic clinical manifestations change with the time of day manner. The manifestations frequently get worse at bedtime or in the early hours of the morning. Flares can be produced by both particular stimuli like allergens and general stimuli like exercising, laughing, irritating exposures, cold air, and respiratory tract infections^1^.

Symptoms and exacerbations occur in severe and uncontrolled cases of chronic asthma despite receiving the maximum amount of standard drug therapy. These cases have type 2 (T2), non-T2, or combined mechanisms-induced chronic airway inflammation^3^. Because of the chronic nature of this inflammatory disease, patients may experience restructuring all air passages, including epithelial apoptotic cell death, proliferation and differentiation of smooth muscle cells, and stimulation of fibroblastic cells contributing to matrix formation. These changes are collectively known as “airway remodeling” and, therefore, can contribute to chronic airway obstruction^4,5^.

Inhaled corticosteroids (ICSs) help decrease airway inflammation, which leads to better clinical asthma results^6^. Biological therapy can be used to enhance the response of the patients to moderate- to high-dose ICSs. The biological therapies that are now approved address particular T2 inflammatory mediators, adding therapeutic value for those with particular asthma characteristics (e.g. eosinophilic or allergic)^7–9^. Yet, certain people with chronic asthma, especially those with non-allergic or non-eosinophilic types, are ineligible for current biologic therapies^10,11^.

Thymic stromal lymphopoietin (TSLP), a cytokine released from epithelial cells, is believed to trigger a number of cell groups and inflammatory pathways implicated in the pathophysiology of asthma. The pathogenesis of both T2 and non-T2 mediated asthma is affected by TSLP, which plays a part in the initiation and maintenance of the airway inflammation^12,13^. The epithelium is the source of TSLP, which is released after its exposure to inhaled epithelial pathogens, including allergens, viruses, and bacteria. By upregulating T2 cytokines, TSLP regulates particular elements of neutrophilic inflammation and activates numerous T2 pro-inflammatory cells. These cells include group 2 innate lymphoid, dendritic, and mast cells^13^. TSLP has also been demonstrated to contribute to airway remodeling. This remodeling is done by fibroblasts and airway smooth muscle proliferation as TSLP increases collagen production^14^.

Tezepelumab, a human monoclonal antibody, attaches to TSLP and blocks binding to its heterodimeric receptors^15,16^. Despite basal values of T2 inflammatory biomarkers, tezepelumab decreased flare-ups dramatically in adults with severe uncontrolled asthma in phase 3 “NAVIGATOR” (NCT03347279) and phase 2b “PATHWAY” (NCT02054130) investigations^15^. To combine the existing data and assess the efficiency and safety of tezepelumab as a treatment for severe uncontrolled asthma in adults, we conducted this systematic review and meta-analysis.

## Methods

We conducted this study and presented our findings in accordance with the preferred reporting guidelines for systematic reviews and meta-analyses (PRISMA) 2020^17^ and Cochrane Handbook of Systematic Reviews of Intervention^18^. In addition, we used PROSPERO to register the protocol for this meta-analysis *(CRD: CRD42021290047).*

### Literature search

We searched Cochrane Central Register of Controlled Trials (CENTRAL), PubMed, Web of Science, clinicaltrial.gov, and Scopus for articles from inception to September 25, 2022, with terms related to asthma and tezepelumab. Supplementary file 1 shows the search strategy we used in detail.

### Eligibility criteria and studies selection

Two independent authors Ramadan Abdelmoez Farahat and Bassant Hassan Shawki, examined the articles to check if they fit our inclusion criteria. We considered randomized controlled studies (RCT) that looked at the clinical efficacy and safety of tezepelumab. In adults with severe, uncontrolled asthma who were either males or females aged ≥ 18. GINA 2012 guidelines define severe uncontrolled asthma despite being treated with long-acting beta-agonists coupled with a medium dose of fluticasone (250–500 g/day via a dry-powder inhaler or equivalent) or high dosage of fluticasone (> 500 g/day via dry-powder inhaler or equivalent) of inhaled glucocorticoids^19^.

Except for RCTs, we excluded all other study designs. Also, we did not include research whose extracted data were unreliable for analysis. Another third author arbitrated any discrepancies between the two authors.

### Quality assessment

The Cochrane risk-of-bias tool for randomized trials (RoB 2) was applied to evaluate the quality of included Randomized clinical trials^20^. The Rob2 tool consists of six domains: (1) the randomization process, (2) missing outcome data, (3) deviations from the intended interventions, (4) selection of the reported result, (5) measure of the outcome and (6) other bias. The response options of the authors were classified as yes, probably yes, probably no, no, and no information. Two authors separately rated the quality, and all the debates were dealt with and resolved.

### Data extraction and study outcomes

Two authors, Ahmed K. Awad and Eman Reda Gad, worked independently to extract data from a pre-defined excel spreadsheet, including the following data: a brief of the clinical trials’ essential characteristics, descriptions of the patients included in the clinical trials, and tezepelumab outcomes related to safety and efficacy. A discussion between the authors solved discrepancies.

### Outcome definition

Treatment efficacy was assessed by annualized asthma exacerbation rate (AERR), change from baseline in pre-dose/pre-bronchodilator (pre-BD) forced expiratory volume in 1 s (FEV1), weekly mean daily. In addition to asthma symptom diary score, ACQ-6 Score, standardized asthma quality of life questionnaire for 12 years and older (AQLQ(S) + 12) total score, European quality of life-5 dimensions 5 level version (EQ-5D-5L) health state evaluation at Week 52, blood eosinophil count, FeNO, and serum total IgE.

Treatment-emergent adverse events (TEAEs) and Treatment-emergent serious adverse events (TESAEs) assessed the occurrence of the adverse events.

### Data synthesis and assessment of heterogeneity

For statistical analyses, we used Revman software Version 5.4.1. For dichotomous data, pooled risk ratio (RR) was used, while for continuous data, the mean difference was used with 95% confidence intervals (CI). We used the random-effect model for the analysis. We considered p-value < 0.05 as a significant point. For heterogeneity, I-square and p-value were used. If the p-value was < 0.05 or I-square was > 60%, the analysis was considered heterogeneous. A leave-one-out test or subgrouping analysis was adopted to solve the heterogeneity^21^.

## Results

### Literature search results

**Figure 1:**
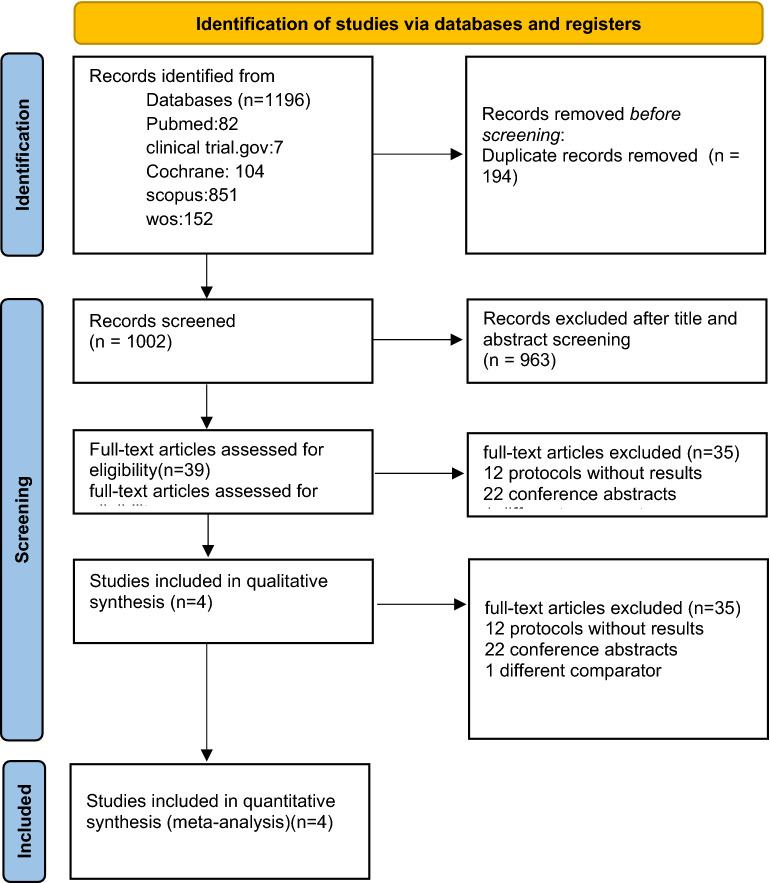
Preferred reporting items for systematic reviews and meta-analyses (PRISMA). From reference^17^. For more information, visit: http://www.prisma-statement.org/.

We obtained 1196 studies from clinical trial.gov, PubMed, Web of Science, and Cochrane library, Scopus. 194 of them were duplicates. After removing the duplicates and the title and abstract screening, 963 articles were excluded as they did not follow our inclusion criteria, while 39 full-text articles were evaluated for eligibility. Finally, the meta-analysis included four RCTs (Fig. 1).

### Summary of the included studies

1600 patients made up the whole sample size of the meta-analysis. 798 patients received tezepelumab, and 802 patients received a placebo. Patients were allowed to use the concomitant medication in the four studies. In contrast, patients were allowed to utilize short-acting beta two agonists (SABA) as rescue medicine, and all participants in the study groups continued to receive inhaled glucocorticoids along with rescue medications that may or may not include oral glucocorticoids without alteration.

A comprehensive overview of the included trials is provided in Table 1.

**Table 1:** Comprehensive overview of the included trials.

Study ID	Study design, country, and timing	Criteria	Sample size	Tezepelumab treatment regimen	Control group	study duration
Diver S. et al. 2021	RCT	1- Male or female, aged 18–75 years, weight ≥ 40 kg at visit 1	Total = 116	Tezepelumab 210 mg (N = 59)	N = 57	2 years
	Canada, Denmark, Germany, United Kingdom, United States	2- Documented physician-diagnosed asthma for ≥ 12 months before visit 1	Tezepelumab = 59			
	Between Nov 2, 2018 and Nov 16, 2020	3- Physician-prescribed asthma controller medication with medium- or high-dose ICS for at least 12 months before visit 1 (as per GINA 2018 guidelines)	Placebo = 57			
		4- Documented use of at least one additional maintenance asthma controller medication (e.g. LABA, LTRA, theophylline or LAMA) for at least 3 months before visit 1			Participants received placebo matched to Tezepelumab dose	
		5- Predicted normal value for morning prebronchodilator FEV1 > 50% and > 1 L at visit 1 or visit 2				
		6- Documented historical FEV1 reversibility of ≥ 12% and ≥ 200 mL in the 12 months before visit 1 or at visit 2				
		7- ACQ-6 score ≥ 1.5 at visit 1 or visit 2				
Menzies-Gow et al. 2021	RCT	1- Age: 12–80	Total = 1059	Tezepelumab 210 mg (N = 528)	N = 531	3 years
		2- Documented physician-diagnosed asthma for at least 12 months				
		3- Subjects who have received a physician-prescribed asthma controller medication with medium or high dose ICS for at least 12 months				
	Argentina, Australia, Austria, Brazil, Canada, France, Germany, Israel, Japan, Korea, Russian Federation, Saudi Arabia, South Africa, Taiwan, Ukraine, United Kingdom, United States, Vietnam	4- Documented treatment with a daily dose of either medium or high dose ICS (≥ 500 µg fluticasone propionate dry powder formulation equivalent to total daily dose) for at least 3 months	Tezepelumab = 528		Participants received placebo matched to Tezepelumab dose	
		5- At least one additional maintenance asthma controller medication is required according to the standard practice of care and must be documented for at least 3 months				
		6- Morning pre-BD FEV1 < 80% predicted normal (< 90% for subjects 12–17 yrs)				
	Between November 23, 2017 and September 8, 2020	7- Evidence of asthma as documented by either: Documented historical reversibility of FEV1 ≥ 12% and ≥ 200 mL in the previous 12 months OR Post-BD (albuterol/salbutamol) reversibility of FEV1 ≥ 12% and ≥ 200 mL during screening	Placebo = 531			
		8- Documented history of at least two asthma exacerbation events within 12 months				
		9- ACQ-6 score ≥ 1.5 at screening and on the day of randomization				
Corren et al. 2017	RCT, Bulgaria, Czechia, Hungary, Israel, Japan, Latvia, Lithuania, Serbia, Slovakia, South Africa, Ukraine, United States	1- Age 18 through 75	Total = 550	Tezepelumab 70 mg (N = 138)	N = 138	
		2- Body mass index (BMI) between 18–40 kg/m^2^ and weight greater than or equal to 40 kg	Tezepelumab = 420	Tezepelumab 210 mg (N = 137)	Participants received placebo matched to Tezepelumab dose	
		3- Documented physician-diagnosed asthma—Subjects must have received a physician-prescribed asthma controller regimen with medium- or high-dose inhaled corticosteroids (ICS) plus long-acting β2 agonist (LABA) -If on asthma controller medications in addition to ICS plus LABA, the dose of the other asthma controller medications (leukotriene receptor inhibitors, theophylline, secondary ICS, long-acting anti-muscarinic (LAMA), cromones, or maintenance oral prednisone or equivalent up to a maximum of 10 mg daily or 20 mg every other day for the maintenance treatment of asthma) must be stable. -Subjects must have a documented history of at least two asthma exacerbation events OR at least one severe asthma exacerbation resulting in hospitalization within the 12 months prior to the first study visit	Placebo = 138	Tezepelumab 280 mg (N = 137)		
Wechsler et al. 2022	RCT, Argentina, Germany, Korea, Poland, Turkey, Ukraine, United States	1- Subjects must have received a physician-prescribed medium- or high-dose ICS as per GINA guidelines for at least 12 months	Total = 150	Tezepelumab 210 mg (N = 74)	N = 76	
		2- Subjects must have received physician prescribed LABA and high dose ICS (total daily dose > 500 μg fluticasone propionate dry powder formulation equivalent) for at least 3 months. The ICS and LABA can be parts of a combination product or given by separate inhalers	Tezepelumab = 74			
		3- Additional maintenance asthma controller medications are allowed according to the standard practice of care, i.e. leukotriene receptor antagonists (LTRAs), theophylline, and long-acting muscarinic antagonists (LAMAs), secondary ICS, and cromones. The use of these medications must be documented for at least three months				
		4- Subjects must have received OCS for the treatment of asthma for at least 6 months prior to screening and on a stable dose of between ≥ 7.5 to ≤ 30 mg (prednisone or prednisolone equivalent) daily or daily equivalent for at least 1 month. The OCS dose may be administered every other day (or different doses every other day); the Average dose over 2 days = The daily dose				
		5- Morning pre-bronchodilator (BD) FEV1 must be < 80% predicted normal	Placebo = 76		Participants received placebo matched to Tezepelumab dose	
		6- Subjects must have evidence of asthma as documented by post-BD (albuterol/salbutamol) reversibility of FEV1 ≥ 12% and ≥ 200 mL (15–30 min after administration of 4 puffs of albuterol/salbutamol), documented either in the previous 12 months				
		7- Subjects must have a history of at least one asthma exacerbation event within 12 months				
		8- Minimum 10 days of compliance with the morning and evening eDiary completion and OCS, ICS, LABA and other asthma controller medications as captured in the eDiary during the 14 days prior to randomization and documented physician-diagnosed asthma for at least 12 months				

Tables 2, 3 provide the baseline characteristics of the patients.

**Table 2:** Baseline characteristics of enrolled patients in each included study.

Study ID	Groups	Number of patients n (%)	Age mean ± SD	Females n (%)	Body mass index (BMI) (kg/m^2^) mean ± SD	Pre-BD FEV1 (L), mean ± SD	ACQ-6 score mean ± SD	ICS, inhaled corticosteroids; n (%)	Blood eosinophil count mean ± SD	Total serum IgE—IU/ml mean ± SD	FeNO (ppb), mean ± SD	Maintenance oral corticosteroid use, n (%)	AQLQ(S) + 12 score mean ± SD
Diver S et al. 2021	Tezepelumab	59	50.4 ± 12.7	39 (66%)	30.6 ± 5.8	2.21 ± 0.69	2.43 ± 0.84	Medium 28 (47%)	302 ± 307	161.1 ± 268.13	33.0 (39.4)	4 (7%)	N/A
								High 31 (53%)			Total = 55		
	Placebo	57	50.4 ± 13.9	26 (46%)	28.4 ± 6.4	2.34 ± 0.54	2.03 ± 0.77	Medium 21 (37%)	272 ± 161	106 ± 0.30	31.2 (19.9)	4 (7%)	N/A
								High 35 (61%)			Total = 56		
Menzies-Gow et al. 2021	Tezepelumab	528	49.9 ± 16.3	335 (63.4%)	28.7 ± 7.1	1.8 ± 0.7	2.8 ± 0.8	Medium 131 (24.8%)	327 ± 293	515.7 ± 959.8	41.4 ± 36.3	49 (9.3%)	3.9 ± 1.0
								High 397 (75.2%)					
	Placebo	531	49.0 ± 15.9	337 (63.5%)	28.3 ± 6.9	1.9 ± 0.7	2.8 ± 0.8	Medium 132 (24.9%)	353 ± 488	614.1 ± 1159.5	46.3 ± 44.7	51 (9.6%)	3.9 ± 1.0
								High 398 (75.0%)					
Corren et al. 2017	Tezepelumab low-dose	138	50.8 ± 12.4	89 (64.5%)	28.3 ± 5.1	1.91 ± 0.67	2.72 ± 0.79	Medium 67 (48.6%)	380 ± 328	323 ± 890	35.6 ± 47.8	N/A	4.17 ± 0.93
								High 71 (51.4%)					
	Tezepelumab medium-dose	137	52.7 ± 12.7	87 (63.5%)	28.5 ± 4.9	1.83 ± 0.58	2.70 ± 0.80	Medium 70 (51.1%)	365 ± 351	484 ± 1402	31.5 ± 29.8	N/A	4.20 ± 0.91
								High 67 (48.9%)					
	Tezepelumab high-dose	137	50.4 ± 12.3	91 (66.4%)	27.6 ± 5.0	1.83 ± 0.57	2.64 ± 0.74	Medium 71 (51.8%)	385 ± 433	358 ± 595	33.3 ± 34.4	N/A	4.08 ± 0.91
								High66 (48.2%)					
	Tezepelumab total	412	51.3 ± 12.4	267 (64.8%)	28.1 ± 5.0	1.86 ± 0.61	2.69 ± 0.77	Medium 208 (50.5%)	367 ± 361	388 ± 1018	33.5 ± 38.1	N/A	4.15 ± 0.92
								High 204 (49.5%)					
	Placebo	138	52.3 ± 11.7	94 (68.1%)	28.5 ± 5.6	1.82 ± 0.59	2.66 ± 0.69	Medium 73 (52.9%)	380 ± 328	475 ± 1272	37.8 ± 39.7	N/A	4.09 ± 0.87
								High 65 (47.1%)					
Wechsler et al. 2022	Tezepelumab	74	53.5 ± 12.1	49 (66.22%)	N/A	N/A	N/A	N/A	N/A	N/A	N/A	N/A	N/A
	Placebo	76	53.4 ± 11.9	45 (59.21%)	N/A	N/A	N/A	N/A	N/A	N/A	N/A	N/A	N/A

**Table 3:** Baseline characteristics of enrolled patients in each included study.

Study ID	Groups	Race							
		White	Black	Asian	American Indian or Alaska native	Hispanic or Latino	Not Hispanic or Latino	Native Hawaiian or other Pacific Islander	Others
Diver S et al. 2021	Tezepelumab	54 (92%)	2 (3%)	2 (3%)	0 (0%)	0 (0%)	0 (0%)	0 (0%)	1 (2%)
	Placebo	55 (96%)	1 (2%)	1 (2%)	0 (0%)	0 (0%)	0 (0%)	0 (0%)	0 (0%)
Menzies-Gow et al. 2021	Tezepelumab	332 (62.9%)	30 (5.7%)	146 (27.6%)	0 (0%)	0 (0%)	0 (0%)	1 (0.2%)	19 (3.6%)
	Placebo	327 (61.6%)	31 (5.8%)	149 (28%)	0 (0%)	0 (0%)	0 (0%)	0 (0%)	23 (4.3%)
Corren et al. 2017	Tezepelumab low-dose	131 (94.9%)	4 (2.9%)	3 (2.2%)	0 (0%)	0 (0%)	0 (0%)	0 (0%)	0 (0%)
	Tezepelumab medium-dose	128 (93.4%)	3 (2.2%)	5 (3.6%)	0 (0%)	1 (0.7%)	0 (0%)	0 (0%)	0 (0%)
	Tezepelumab high-dose	122 (89.1%)	6 (4.4%)	5 (3.6%)	0 (0%)	2 (1.5%)	0 (0%)	0 (0%)	2 (1.5%)
	Tezepelumab total	381 (92.5%)	13 (3.1%)	13 (3.1%)	0 (0%)	3 (0.7%)	0 (0%)	0 (0%)	2 (0.5%)
	Placebo	123 (89.1%)	6 (4.3%)	6 (4.3%)	0 (0%)	1 (0.7%)	0 (0%)	0 (0%)	2 (1.4%)
Wechsler et al. 2022	Tezepelumab	62 (83.8%)	1 (1.3%)	11 (14.9%)	0 (0%)	10	64	0 (0%)	0 (0%)
	Placebo	64 (84.2%)	0 (0%)	11 (14.4%)	0 (0%)	14	62	0 (0%)	1

### Quality assessment

**Figure 2:**
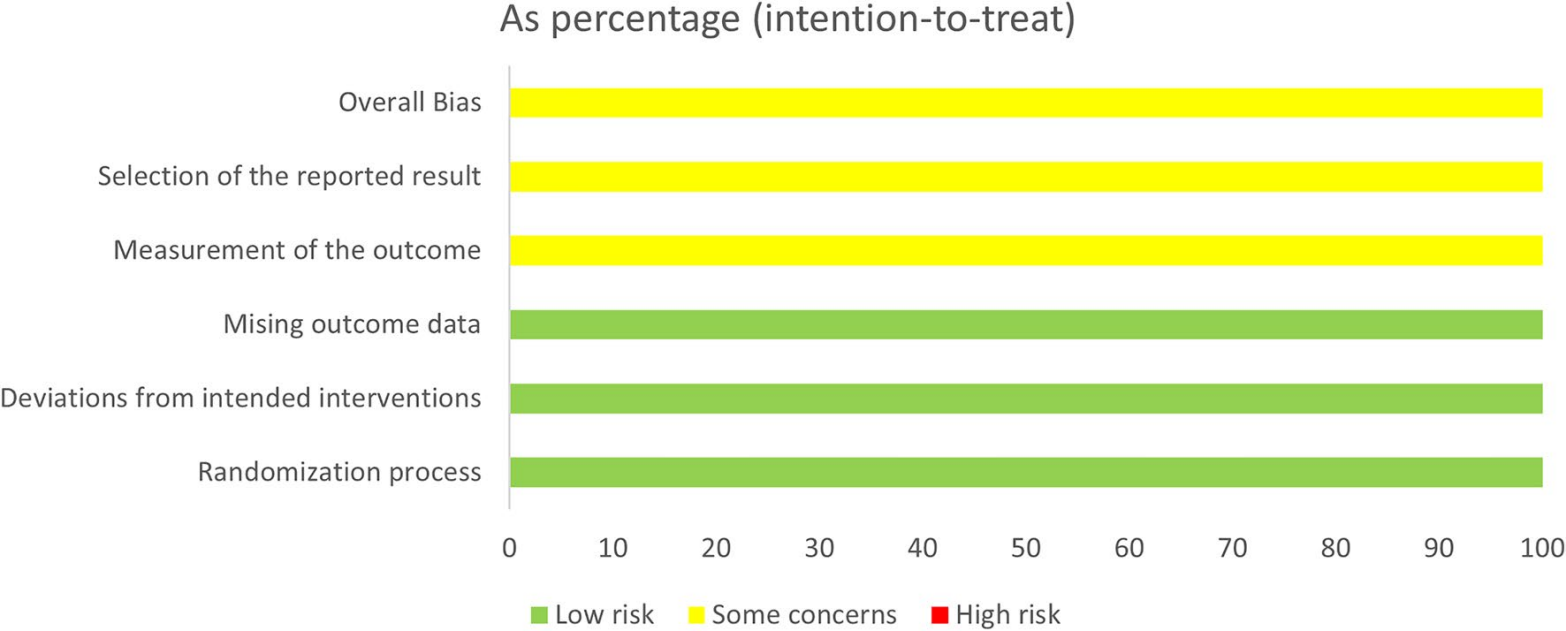
Risk of bias graph for randomized controlled trials using ROB2.

**Figure 3:**
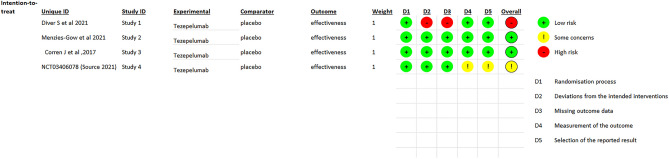
Risk of bias summary for randomized controlled trials using ROB2.

ROB 2 tool evaluated the bias risk of the included trials from low to high risk. Figures 2, 3 illustrate the bias risk summary.

Randomization process bias: We evaluated all the included trials as low risk for the randomization process.

Intended interventions bias: In terms of deviations from the intended interventions, the majority of the included trials showed a low risk of bias except for Diver S. et al. 2021, which were judged as high risk. This is because the statistical analysis that was done to calculate the impact of assignments was as treated analysis, and there was a loss during the follow-up exceeding 5% of the population.

Missing outcome data bias: Due to the use of the intention to treat analysis, most included trials had a low risk of bias in the missing outcome data, except Diver S et al. 2021. which had a high risk of bias because the authors used an as-treated analysis with 8% withdrawal in the intervention group, and they did not mention the reasons for exclusion in the intervention group.

Measurement outcome bias: Because all outcome assessors were blinded and used appropriate outcome measurement methods, we judged the bias risk in the measurement of the outcome as low in the majority of the included trials. Hence, due to the lack of information about blinding the outcome assessor, we judged Wechsler 2022 as having some concerns.

Selection of the reported results bias: We judged the risk of bias owing to the selection of the reported results of Wechsler 2022 as raised some concerns, but the other trials were rated low risk because all outcomes established in the results were in the protocols.

Other Bias: There is no other bias.

### Publication bias

We couldn’t use Egger’s test for funnel plot asymmetry in this study to detect publication bias as we have only four studies, and for less than ten pooled studies, publication bias assessment is unreliable^22^.

### Data-analysis

#### Annualized asthma exacerbation rate (AAERR)

**Figure 4:**
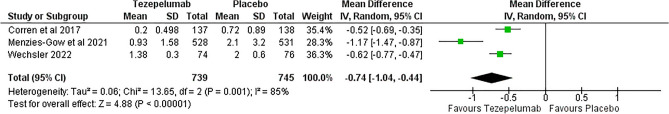
Annualized asthma exacerbation rate (AAERR).

Our analysis of annualized asthma exacerbation rate (AERR) includes three studies with a total of 739 patients in the tezepelumab arms and 745 patients in placebo arms, revealed a significant decrease in AERR favoring tezepelumab with MD -0.74 (95% CI [− 1.04, − 0.44], p < 0.00001). High heterogeneity was observed (p = 0.001, I^2^ = 85%) but we could not perform leave one out test as we have only three studies in the analysis. (Fig. 4).

#### Change from the baseline in Pre-dose/pre-bronchodilator (pre-BD) forced expiratory volume in 1 s (FEV1)

**Figure 5:**
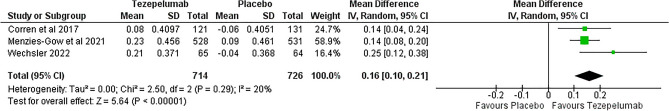
Change from the baseline in Pre-dose/pre-bronchodilator (pre-BD) forced expiratory volume in 1 s (FEV1).

Analysis of pre-BD FEV1 includes three studies with a total of 714 patients in the tezepelumab arms and 726 patients in placebo arms, revealed a significant increase in FEV-1 favoring tezepelumab with MD 0.16 (95% CI [0.10, 0.21], p < 0.00001). Low heterogeneity was found (p = 0.29, I^2^ = 20%) (Fig. 5).

#### Change from baseline in asthma control questionnaire-6 (ACQ-6) score

**Figure 6:**
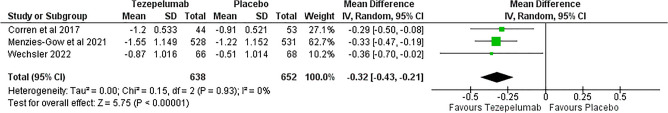
Change from baseline in Asthma Control Questionnaire-6 (ACQ-6) Score.

Analysis of ACQ-6 score includes three studies with a total of 638 patients in the tezepelumab arms and 652 patients in placebo arms, revealed a significant decrease in ACQ-6 score favoring tezepelumab with MD − 0.32 (95% CI [− 0.43, − 0.21], p < 0.00001). Heterogeneity evidence was found (p = 0.93, I^2^ = 0%) (Fig. 6).

#### *Change from baseline in standardized asthma quality of life questionnaire for 12 years and older (AQLQ* + *12) total score*

**Figure 7:**
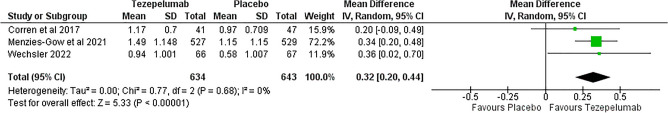
Change from baseline in Standardized Asthma Quality of Life Questionnaire for 12 years and older (AQLQ + 12) total score.

Analysis of AQLQ + 12 total score includes three studies with a total of 634 patients in the tezepelumab arms and 643 patients in placebo arms, revealed a significant increase in AQLQ + 12 score favoring tezepelumab with MD 0.32 (95% CI [0.20, 0.44], p < 0.00001). Heterogeneity evidence was found (p = 0.68, I^2^ = 0%) (Fig. 7).

#### Change from the baseline in blood eosinophil count

**Figure 8:**
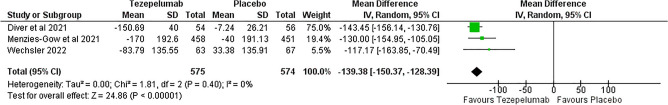
Change from the baseline in Blood eosinophil count.

Analysis of blood eosinophil count includes three studies with a total of 575 patients in tezepelumab arms and 574 patients in placebo arms, which revealed a significant decrease in blood eosinophil count favoring tezepelumab with MD -139.38 cells/mcL (95% CI [− 150.37, − 128.39], p < 0.00001). Heterogeneity evidence was found (p = 0.40, I^2^ = 0%) (Fig. 8).

#### Change from the baseline in FeNO

**Figure 9:**
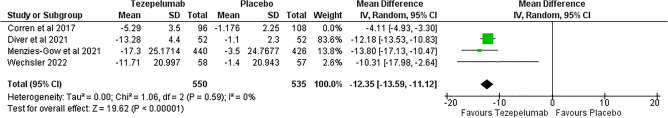
Change from the baseline in FeNO.

Analysis of FeNO levels includes four studies with a total of 646 patients in the tezepelumab arms and 643 patients in placebo arms, which revealed a significant decrease in FeNO levels favoring tezepelumab with MD − 10 ppb (95% CI [− 15.81, − 4.18], p = 0.0008). High heterogeneity was observed (p < 0.00001, I^2^ = 97%), which was solved by sensitivity analysis excluding Corren et al. 2021 (p = 0.59, I^2^ = 0%) (Fig. 9).

#### Change from the baseline in serum total IgE

**Figure 10:**
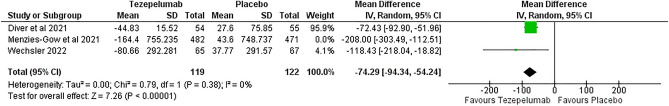
Change from the baseline in Serum IgE.

Analysis of serum total IgE includes three studies with a total of 601 patients in the tezepelumab arms and 593 patients in placebo arms, revealed a significant decrease in serum total IgE favoring tezepelumab with MD -123.51 UI/ml (95% CI [− 206.52, − 40.50], p = 0.004). Low heterogeneity was found (p = 0.38, I^2^ = 0%) (Fig. 10).

#### Adverse effects

**Figure 11:**
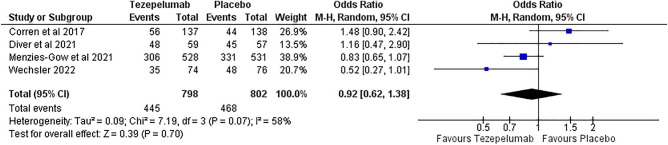
Any serious adverse effects outcome.

**Figure 12:**
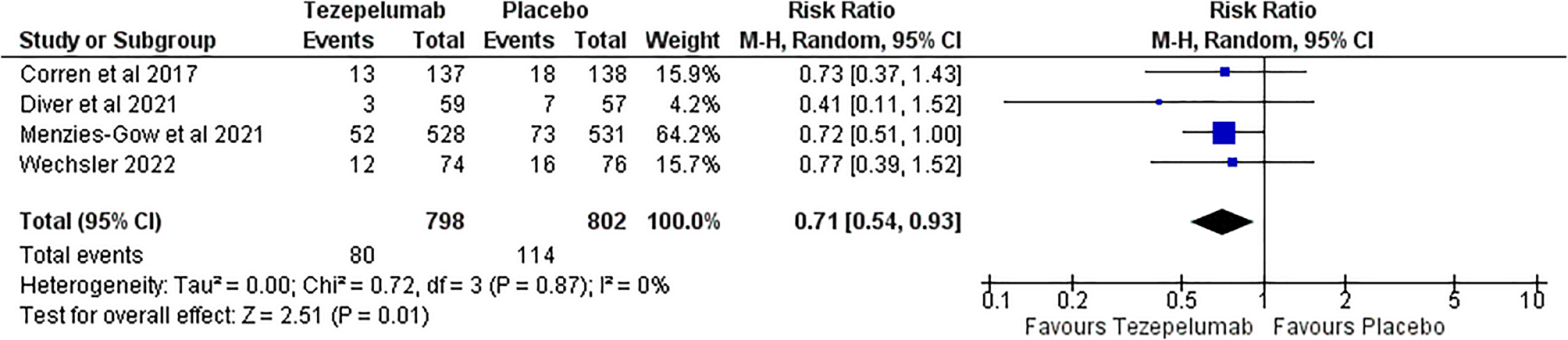
Any adverse effects outcome.

Tezepelumab significantly lowers the risk of any serious adverse effects than placebo, with RR 0.71 (95% CI [0.54, 0.93], p = 0.01), as opposed to the analysis of any adverse effects showing no significant risk reduction between tezepelumab and placebo with RR 0.92 (95% CI [0.62, 1.38], p = 0.70). (Figs. 11, 12).

## Discussion

This study revealed significant improvements in asthma management, lung functional status, well-being, and quality of life with tezepelumab treatment compared to placebo. Tezepelumab significantly decreased the occurrence of asthma exacerbations in adults with chronic uncontrolled asthma, including those with reduced blood eosinophil levels, compared to placebo. In addition, tezepelumab showed significant improvements in ACQ-6, AQLQ(S) + 12 scores, and FEV1, decreasing hospitalization or emergency room visits. However, the tezepelumab and placebo groups did not differ significantly regarding the frequency and kinds of adverse incidents.

Tezepelumab concurrently decreased blood eosinophil count, FENO, and serum total IgE levels, indicating that the drug inhibits numerous inflammatory pathways. Tezepelumab influence on these biomarker levels could be linked to the lower levels of interleukin-5 and interleukin-13^23^. The observed decrease in serum total IgE levels could be related to lower levels of interleukin-4 and interleukin-13, which would result in a gradual reduction in B-cell shifting from IgM to IgE isotype production. These findings support the theory that TSLP inhibition has a more considerable physiological impact than just targeting individual T2 cytokines^24^.

In response to stimulation (including irritants, infections, harmful airborne particles, and traumatic agents), the function of TSLP as an early mediator between cells of immunity and epithelial cells of the airways indicates that tezepelumab may normalize local inflammation through allergic and non-allergic mechanisms, regardless of blood eosinophil count. It is anticipated that TSLP inhibition will reduce the T2 cytokine produced by T-memory cells, mast cells, and innate lymphocyte type 2 cells across the spectrum of inflammation. The roles of TSLP in triggering responsiveness via dendritic cells and interactions between mast cells and smooth muscle cells of the air passages are pathways that could be important to inflammation in low-eosinophil populations^13,25,26^.

According to a previous systematic review, omalizumab, tezepelumab, and dupilumab may modulate airway hyperresponsiveness by direct action on smooth muscle cells in the airway, in addition to indirect effects on parasympathetic activity and eosinophilic inflammation^27^. Another worldwide study on adolescents and adults showed that tezepelumab reduced the annual occurrence of asthma symptoms significantly in adults and adolescents with severe uncontrolled asthma, even in individuals who have blood eosinophil counts as low as 300 cells per microliter at baseline^28^. Furthermore, another trial, “The PATHWAY” (NCT03347279), showed more reduction in the asthma symptoms yearly incidence than “The NAVIGATOR” trial (NCT02054130).

Tezepelumab, as compared to placebo, decreased exacerbations in patients who have or who do not have perennial allergy in a 52-week trial. Furthermore, lung function was enhanced, and blood eosinophil counts and FENO levels decreased regardless of allergy status. In this study, no significant differences were found in the majority of asthma severity assessments between individuals who have and who do not have allergies at baseline. However, there were some differences in the biological indicators of the inflammatory process: patients who have allergic reactions had greater serum total IgE and high FENO at baseline, without discernible change in plasma eosinophil count. Rhinitis and atopic dermatitis were also more prevalent in allergy patients than in non-allergy patients and younger ones^29^.

Patients who took tezepelumab instead of a placebo had a more significant percentage of responders as evaluated by the AQLQ(S) + 12 and ACQ-6^30^. The percentage of placebo patients whose ACQ-6 and AQLQ(S) + 12 scores increased by clinically significant levels was between 61–78% and 70%, respectively. This finding is consistent with evidence from studies investigating other biologic therapies in asthma^31–34^. The high number of placebo group responders in these studies might be attributed to greater adherence to standard-of-care medicines while participating in these clinical trials. Furthermore, several trials have demonstrated that a patient’s impression of the benefits of clinical trial participation may result in a positive response to placebo therapy^35,36^.

Tezepelumab did not decrease submucosal neutrophil cells or lymphocyte cells of the airways, which is reassuring from a safety standpoint. Eosinophil-depleting medications do not cause clinically significant immunosuppressive response, as evidenced by the fact that there was no increased incidence of infectious events in the tezepelumab compared to placebo groups in the previous trial^37^. Also, Corren et al. found that tezepelumab was safe, the number of patients was modest, and treatment was administered every 2 or 4 weeks for a total duration of 1 year^15^.

## Strengths and limitations

Tezepelumab efficacy and safety in patients with severe uncontrolled asthma were summarized in this systematic review and meta-analysis. The study includes four RCTs, yielding a high level of evidence. The studies included ranged in quality from poor to excellent. The study limitations were due to the inherent research: RCTs have often been conducted in small, carefully selected groups of asthmatic patients. Furthermore, the majority of the identified heterogeneity was not resolved. Moreover, due to the small number of papers included, publication bias could not be examined. In addition, we could not get data of Wechsler 2022 from its full text, so we got its data from the protocol.

## Conclusion

Tezepelumab provided considerable ability to control the exacerbations of severe uncontrolled adult asthmatics. However, minimal is known regarding the actual clinical impact of monoclonal antibodies like tezepelumab in the treatment of asthma. Further research involving large, ethnically varied samples of individuals with uncontrolled asthma is critical to address this clinical challenge for long-term illness care.

## Supplementary Information

Supplementary Information.

## Data Availability

All data generated or analyzed during this study are included in this published article or in the data repositories listed in references.

## Abbreviations

TSLP: Thymic stromal lymphopoietin
AAERs: Annualized asthma exacerbation rates
GINA: The global initiative of asthma
ICSs: Inhaled corticosteroids
PRISMA: The preferred reporting items for systematic reviews and meta‐analyses
CENTRAL: Cochrane central register of controlled trials
RCT: Randomized controlled trials
pre-BD: Pre-dose/pre-bronchodilator
FEV1: Forced expiratory volume in 1 s
ACQ-6 score: Asthma control questionnaire-6 score
AQLQ(S) + 12 total score: Standardized asthma quality of life questionnaire for 12 years and older total score
EQ-5D-5L: European quality of life-5 dimensions 5 level version
TEAEs: Treatment-emergent adverse events
TESAEs: Treatment-emergent serious adverse events
SABA: Short-acting beta two agonists
MD: Mean difference
