# Supplementary material for: Safety and efficacy of tezepelumab vs. placebo in adult patients with severe uncontrolled asthma: a systematic review and meta-analysis
Source: Sci Rep. 2022 Dec 3;12:20905. doi: 10.1038/s41598-022-24763-9 (PMC9719466; doi:10.1038/s41598-022-24763-9)
Supplement: Supplementary file 1 — Supplementary Information. [file 41598_2022_24763_MOESM1_ESM.docx]

**Safety and efficacy of tezepelumab vs placebo in adult patients with severe uncontrolled asthma: a systematic review and meta-analysis.**

**Mahmoud Shaban Abdelgalil^1^ , Asmaa Ahmed Elrashedy^2^,Ahmed K. Awad^1^ ,Eman Reda Gad^3^ , Mahmoud M. Ali^4^,Ramadan Abdelmoez Farahat^2^,Bassant Hassan Shawki^1^, Mohamed Abd-ElGawad^5*^**

1. *Faculty of Medicine, Ain-shams University, Cairo, Egypt.*
2. *Faculty of Medicine, Kafrelsheikh University, Kafrelsheikh, Egypt.*
3. *Faculty of Medicine, Cairo University, Cairo, Egypt.*
4. *Faculty of Pharmacy, Al-Azhar University, Assiut, Egypt.*
5. *Faculty of Medicine, Fayoum University, Fayoum, Egypt.*

**Corresponding author*:**

Faculty of Medicine, Fayoum University, Fayoum, Egypt.

Postal address; 5 Al-Touba Street, from Al-Fanya Street, Al-Hadka road, Fayoum, Fayoum, Egypt

Email: mm2953@fayoum.edu.eg

ORCID: 0000-0003-4120-9161

Phone: +201069401202

**Search strategy: -**

**PubMed**;

(Tezepelumab OR AMG-157 OR MEDI-9929 OR MEDI-19929 OR MEDI9929) AND (Uncontrolled Asthma OR Uncontrolled Bronchial Asthma OR Uncontrolled Bronchial Asthmas OR Asthma )

No limitations were applied.

From inception to September 25, 2022

Results: 82

**Cochrane:**

(Tezepelumab OR AMG-157 OR MEDI-9929 OR MEDI-19929 OR MEDI9929) AND (Uncontrolled Asthma OR Uncontrolled Bronchial Asthma OR Uncontrolled Bronchial Asthmas OR Asthma)

No limitations were applied.

From inception to September 25, 2022

Results: 104

**Scopus:**

ALL ( ( "Tezepelumab" OR " AMG-157" OR "MEDI-9929" OR "MEDI-19929" OR "MEDI9929" ) AND ( "Uncontrolled Asthma" OR " Uncontrolled Bronchial Asthma" OR "Uncontrolled Bronchial Asthmas" OR "Asthma" ) )

No limitations were applied.

From inception to September 25, 2022

Results: 851

**Web of Science:** (Tezepelumab OR AMG-157 OR MEDI-9929 OR MEDI-19929 OR MEDI9929) AND (Uncontrolled Asthma OR Uncontrolled Bronchial Asthma OR Uncontrolled Bronchial Asthmas OR Asthma)

No limitations were applied.

From inception to September 25, 2022

Results: 152

**Clinical trial .gov :**

Tezepelumab AND Uncontrolled Asthma

No limitations were applied.

Results: 7
